# Emerging problem in medical education: ancillary staff and order sets

**DOI:** 10.5116/ijme.58e8.fd5d

**Published:** 2017-04-21

**Authors:** Ali Motabar, Van La, Daniel Kim

**Affiliations:** 1 Department of Internal Medicine, University of California, Riverside, School of Medicine, Riverside, CA, USA

**Keywords:** Residency training, electronic medical record, order sets, hospital ancillary staff, physician computerized order entry

## Introduction

Hospitals are constantly trying to improve quality of patient care by increasing compliance with the core measures required by the Centers for Medicare and Medicaid Services, a federal organization that administers the Medicare and Medicaid programs (health insurances provided by the federal government of the United States for people age 65 or older and those with limited income, respectively). The agency also tracks a variety of evidence-based and scientifically-researched standards of care that have been shown to result in improved clinical outcomes.^1^ The ending result is that the US health care system will become more outcome-driven and cost-effective. To reach these goals of providing more accountable care to the patients, many hospitals establish various teams that are comprised of specialty nurses and nurse practitioners for the management of wound care, nutrition, respiratory care, rapid response (RRT), sepsis, diabetic care, and code blue (a national communication cue among hospitals in the United States to gather physicians and other supports responding to a patient with cardiopulmonary arrest).

### Challenge in Current Medical Education

With the implementation of electronic health records in the United States as well as internationally, hospitals create patient care order sets (also known as computerized provider order entry internationally; these are treatment protocols or collections of orders that are organized into checklist based on evidence-based guidelines) for hospital admissions and/or commonly admitted medical conditions.^2^^,^^3^ Commonly seen order sets such as stroke, chest pain, diabetic ketoacidosis (DKA), congestive heart failure (CHF), etc. are established for the purpose of standardizing management approach. Despite improvement in standard compliance and patient outcomes through the implementation of ancillary staffs and order sets, it seems that the roles of physicians are becoming more passive. Furthermore, the over-dependence on these services may be a detriment to the residents' education and learning.

Ancillary support teams are now available in many hospitals throughout the nation. For instance, RRT was initiated as a result of the “100,000 Lives Campaign” in 2005 aiming to reduce morbidity and mortality in hospital setting.^4^^,^^5^ Residents at teaching hospitals are more likely to take passive roles in evaluation and management of the patients with the presence of these teams. This is especially important where physicians in training should directly be involved in the evaluation and treatment of their patients. There is no doubt that these well-trained teams have the best experience in assessing, documenting, and managing conditions like diabetes, wound, sepsis, etc. appropriately and result  in better outcomes and compliance with hospital goals. Nonetheless, this may come with the unintended result of residents being less involved in direct patient care.

In a recent survey of the current post-graduate year one to three physician residents of the internal medicine residency program at Riverside University Health System Medical Center (RUHSMC), only four of the thirty-six residents had the opportunity to lead a code blue. The lack of hands-on experience with directing a code blue is likely due to the availability of the code blue team at the hospital, though residents have been receiving periodic mock code sessions as part of their simulation lab training.

Multiple large-scale studies have clearly documented the benefits of adopting order sets in medical practices to “improve the quality and efficiency of patient care, reduce errors, and increase adherence to evidence-based care guidelines”.^2^ However, the impact of implementing these order sets has on physicians’ critical thinking skills are yet to be analyzed. By providing complex patient care via a simple checklist, this may as well encourage the habit of “thinking inside the box”, and may have potential impact on the future advancement of medicine, in which the majority is based upon creativity as the result of comprehensive medical reasoning. To further explore the potential negative impact of order sets on the medical education of residents, a literature review was performed via the PubMed website.

Only one out of the three relevant resulted articles directly measured the effect of order sets on medical education of residents. Yu and colleagues found that there was a statistically significant improvement in post-rotation testing score among the residents who were assigned to the order set group compared to the residents who were assigned to group without order set.^3^ The major limitations of the study were very small sample size and the baseline academic performance characteristics of these residents in each study group were not assessed prior to the study. More studies are needed in assessing various areas of residency education with the implementation of order sets and ancillary staff.

## Conclusions

It seems that increasing segmentation of care and pre-made order sets may, overall, threaten the autonomy of the training physicians. Furthermore, this so-called “convenience” may limit their opportunities to truly understand and develop skills to take the appropriate interventions without the system of safety nets. This is an area that needs more attention and appropriate intervention.

### Conflict of Interest

The authors declare that they have no conflict of interest.
